# NoVaTeST: identifying genes with location-dependent noise variance in spatial transcriptomics data

**DOI:** 10.1093/bioinformatics/btad372

**Published:** 2023-06-07

**Authors:** Mohammed Abid Abrar, M Kaykobad, M Saifur Rahman, Md Abul Hassan Samee

**Affiliations:** Department of Computer Science and Engineering, Brac University, Dhaka 1212, Bangladesh; Department of Computer Science and Engineering, Brac University, Dhaka 1212, Bangladesh; Department of Computer Science and Engineering, Bangladesh University of Engineering and Technology, ECE Building, Palashi, Dhaka 1205, Bangladesh; Department of Integrative Physiology, Baylor College of Medicine, Houston, TX 77030, United States

## Abstract

**Motivation:**

Spatial transcriptomics (ST) can reveal the existence and extent of spatial variation of gene expression in complex tissues. Such analyses could help identify spatially localized processes underlying a tissue’s function. Existing tools to detect spatially variable genes assume a constant noise variance across spatial locations. This assumption might miss important biological signals when the variance can change across locations.

**Results:**

In this article, we propose *NoVaTeST*, a framework to identify genes with location-dependent noise variance in ST data. NoVaTeST models gene expression as a function of spatial location and allows the noise to vary spatially. NoVaTeST then statistically compares this model to one with constant noise and detects genes showing significant spatial noise variation. We refer to these genes as “noisy genes.” In tumor samples, the noisy genes detected by NoVaTeST are largely independent of the spatially variable genes detected by existing tools that assume constant noise, and provide important biological insights into tumor microenvironments.

**Availability and implementation:**

An implementation of the NoVaTeST framework in Python along with instructions for running the pipeline is available at https://github.com/abidabrar-bracu/NoVaTeST.

## 1 Introduction

Spatial variation in gene expression is strongly linked to tissue function and physiology (Longo *et al.* 2021, Rao *et al.* 2021, Moses and Pachter 2022, Palla *et al.* 2022). Although the first demonstrations of this phenomenon came from low-throughput techniques, spatial transcriptomics (ST) now allows transcriptome-scale identification of genes that have location-dependent expression. The commercially available Visium ST, e.g. captures the transcriptome of a tissue slice in a spatial grid of spots, each spot containing 5–15 cells on average (Moses and Pachter 2022).

Modeling the gene expression as a function of location is the first step toward the spatial variation analysis of ST data. Existing models of these data assume homoscedasticity, i.e. gene expression noise is assumed to have constant variance across locations. Thus, a gene’s expression $y_{i}$ at location $x_{i}$ is modeled using a function $f(x_{i})$ and a Gaussian noise ϵ with mean zero and variance $\sigma^{2}$:


$$y_{i}=f(x_{i})+\epsilon ,\;\text{where}\;\epsilon ∼N(0,\;\sigma^{2}).$$

These models have identified genes with significant location-dependent expression changes, providing valuable biological insights (Svensson *et al.* 2018, Sun *et al.* 2020). However, we still lack models that assess if the noise variance of a gene’s expression is location dependent, i.e. heteroscedastic (McCulloch 1985).

**Figure 1:**
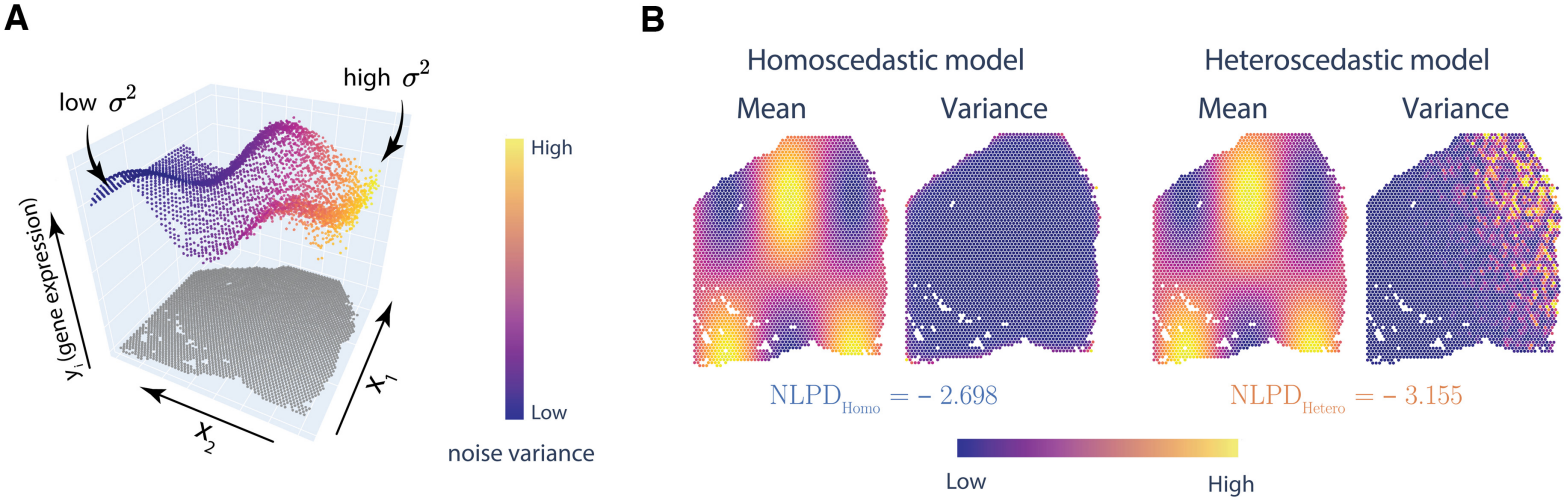
Simulated example to show the importance of heteroscedastic modeling. (A) Simulated gene expression with heteroscedastic noise. The noise variance increases as $x_{1}$ decreases and $x_{2}$ increases. The coordinates are taken from the Visium data of a tissue sample with 3650 spots. (B) Predicted mean and noise variance along with the NLPD of the simulated data for spatial models—homoscedastic and heteroscedastic. A lower NLPD indicates a better model fitting.

To capture the changes in noise variance, the underlying model of gene expression as a function of spatial coordinates must incorporate heteroscedasticity. In this case, the variance of ϵ is not an unknown constant $\sigma^{2}$ but a variable $\sigma_{i}^{2}$ that depends on the location $x_{i}$. However, using a variance parameter for each location could lead to a very large number of parameters, which could potentially lead to overfitting. Instead, $\sigma_{i}^{2}$ is modeled using another function of location $g(x_{i})$. Fig. 1A shows an example of a simulated gene expression with smooth mean function and heteroscedastic noise, i.e. $\sigma_{i}^{2}$ changing with $x_{i}$. In the presence of heteroscedastic noise, a homoscedastic (constant noise) model would not be able to capture the spatial variation of noise, and thus a heteroscedastic model is expected to yield a better fit. One way to measure this goodness of fit is to use negative log predictive density (NLPD) on a test dataset. Studies in heteroscedastic modeling commonly use NLPD to compare models (Le *et al.* 2005, Kersting *et al.* 2007, Lázaro-Gredilla and Titsias 2011, Zhang *et al.* 2020) instead of mean-squared error (MSE) or variance-normalized MSE because NLPD penalizes both over-confident and under-confident predictions (Kersting *et al.* 2007). As an example, the NLPDs of a homoscedastic model and a heteroscedastic model for a simulated expression are shown in Fig. 1B. The homoscedastic model fails to capture spatially variable noise, while the heteroscedastic model predicts noise variance, resulting in lower NLPD.

The premise of the existence of heteroscedastic noise is set by prior analyses of spatial data in biology (Bradley and Pollard 2017, Park *et al.* 2019, Blackledge *et al.* 2020), economics (Brooks *et al.* 2001, Liu 2004, Brownlees *et al.* 2011), and robotics (Vasudevan *et al.* 2010, Bauza and Rodriguez 2017, Smith *et al.* 2018). For example, the magnitude of imaging noise for apparent diffusion coefficient during whole-body diffusion-weighted MRI is heteroscedastic (Blackledge *et al.* 2020). Another study used heteroscedastic noise variance to encode confidence levels in predicting the tumor mutation burden from whole slide images (Park *et al.* 2019). In economics, heteroscedastic regression is used for modeling time-varying volatility and stochastic volatility models in time series data (Brownlees *et al.* 2011). Heteroscedastic regression is also used in robotics (Bauza and Rodriguez 2017, Smith *et al.* 2018), including where multimodal sensor data are combined for terrain modeling (Vasudevan *et al.* 2010). Other fields where heteroscedastic noise variance is used include vehicle control (Lázaro-Gredilla *et al.* 2014), biophysical variable estimation (Kou *et al.* 2015), and cosmological redshift estimation (Almosallam *et al.* 2016).

Given the above precedent, we posit that spatial signals may not always indicate constant variance, and thus suggest modeling ϵ of gene expression with a location-dependent variance $\sigma_{i}^{2}$. Motivated by this, we propose ***no****ise* ***va****riation* ***te****sting in* ***ST****data (NoVaTeST)*, a pipeline to identify genes with statistically significant heteroscedasticity. NoVaTeST offers a more generalized framework for modeling gene expression with spatial coordinates that takes heteroscedasticity into account. It then compares the NLPDs using rigorous statistical tests to select the best model (homoscedastic or heteroscedastic) for each gene to find the ones with location-dependent noise. NoVaTeST also clusters the detected genes with similar noise variance patterns.

In the context of ST data, spatial variation of noise variance could indicate gene expression variation due to sequencing technology, as well as variation due to biology. For example, a common source of technical noise is the mean–variance relation (Anders and Huber 2010, Nakagawa and Schielzeth 2012), where the noise variance typically increases with the mean expression of a gene. Several variance-stabilizing transformations are available to remove this type of technical noise (Anscombe 1948, Eling *et al.* 2018). On the other hand, the variation in noise could also be due to underlying biologies, such as cell-type heterogeneity (Li *et al.* 2022), cell-abundance variation, and phenotypical variation across spatial coordinates. Moreover, if the sample being analyzed is partially affected by some condition, the noise variance for some genes is likely to be different in the affected region compared to the nonaffected region (Dinalankara and Bravo 2015). The exact cause of heteroscedasticity will depend on the sample being analyzed and requires additional information from other data modalities such as single-cell RNA-seq data, spatial metabolomics data, etc.

## 2 Materials and methods

The different components of the NoVaTeST pipeline are described in the following sections. We first describe the ST data used to test the pipeline, followed by the representation of the ST data. Then, we describe the spatial modeling of the ST data—both the homoscedastic and the heteroscedastic models. Next, we describe the statistical model selection technique used to identify genes with spatially variable noise. Finally, we describe the method for clustering the genes based on their spatially variable noise.

### 2.1 Data source and QC filtering

We applied our NoVaTeST pipeline on two different ST datasets. The first one is a 10x Visium data downloaded from the study of Ji *et al.* (2020) (GEO: GSE144239), which contains single-cell transcriptomics data (scRNA-seq) (GEO: GSE144240) as well as ST data from patients with squamous cell carcinoma. The samples are skin tissue slices with squamous cell carcinoma along with patient-matched normal adjacent skin samples. We used the filtered ST data of patient 6 replicate 1 provided by the author (from the file GSE144239_ST_Visium_counts.txt.gz), which contained gene expression counts from 17 736 genes expressed across 3650 spots. Additionally, we filtered out the ERCC genes and mitochondrial genes, as well as practically unobservable genes that had a total count of <3 across all the spots. After filtering, we were left with an expression of 15 725 genes across 3650 spots. The scRNA-seq data contains cell-type annotations of each cell. We applied the FindAllMarkers function of Seurat (Satija *et al.* 2015) on these annotations to obtain the differentially expressed genes for every cell type. The top 20 differentially expressed genes (in terms of the log-fold change) for each cell type were taken as its marker.

The second dataset was downloaded from the Spatial Research website from the study of Thrane *et al.* (2018), which contains ST technology (Ståhl *et al.* 2016) data from patients with stage III cutaneous malignant melanoma. We used the data of patient 1 replicate 1 (from the file ST_mel1_rep1_counts.tsv), which contained gene expression counts from 15 666 genes expressed across 279 spots. Similar to the previous dataset, we filtered out the ERCC and mitochondrial genes, as well as practically unobservable genes that had a total count of <3. After filtering, we were left with gene expression data of 13 088 genes across 279 spots for a melanoma lymph node biopsy sample with three distinct regions—melanoma, stroma, and lymphoid.

### 2.2 Expression data representation

**Figure 2:**
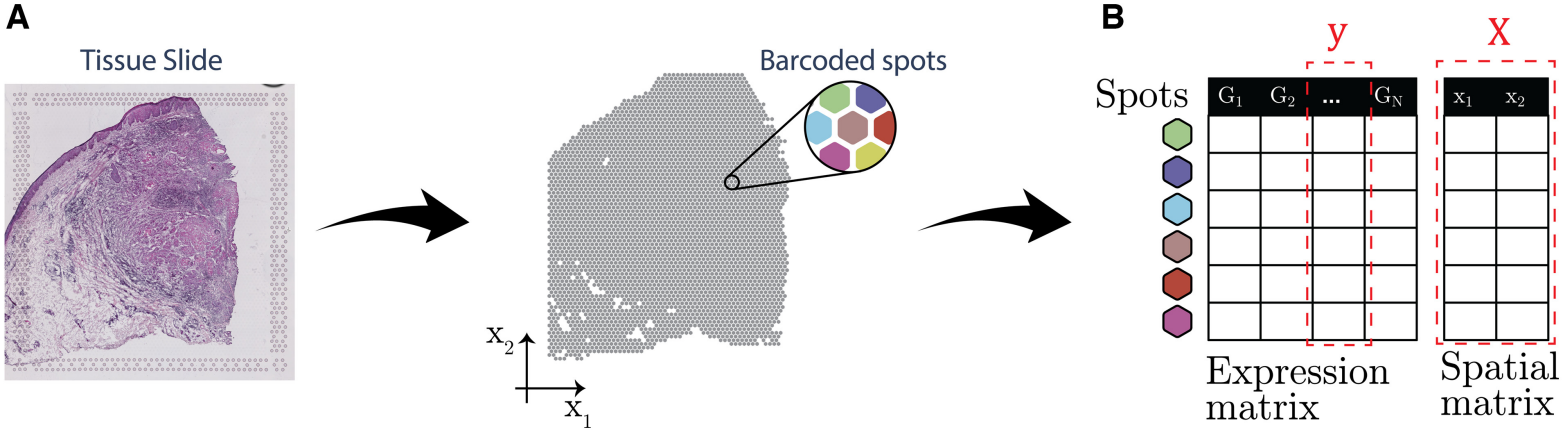
ST data acquisition pipeline for Visium and ST technology. (A) Tissue slice placed on top of a slide to acquire ST data at pre-designated spatially barcoded spots. (B) Representation of ST count data. Each column in the expression matrix represents the expression of a particular gene with spot locations given by the spatial matrix. The expression matrix is a N×G matrix, where *N* is the number of spots and *G* is the number of genes. The spatial matrix is a N×2 matrix, where each row represents the $x_{1}$ and $x_{2}$ coordinates of a spot.

Both ST technology (Ståhl *et al.* 2016) and Visium use barcoded microarrays to capture and sequence mRNA from a tissue sample placed on top of a slide (Fig. 2A). The barcodes attached to each mRNA molecule can be used to map its location back to a specific spot on the slide, where one spot contains the mRNA from 1 to 100 cells. After mapping the reads to a reference genome and grouping the reads by their unique molecular identities (UMIs), the gene expression counts can be obtained for each spot. The resulting data are represented as a matrix where each row represents a spot and each column represents a gene (Fig. 2B). Additionally, the data also contain a matrix of spatial coordinates for each spot, where each row represents a spot and each column represents the horizontal ($x_{1}$) and vertical ($x_{2}$) coordinates of the spot (Fig. 2B). The UMI count data are normalized by the library size of each spot, and then variance is stabilized using the Anscombe technique (Anscombe 1948).

The locations of the spots of the resulting data are denoted as $X={\left[x_{1},\;x_{2},\;\ldots ,\;x_{N}\right]}^{T}$, where $x_{i}=[x_{i_{1}},x_{i_{2}}]$. We denote the normalized and variance stabilized gene expression profile for a given gene as $y={\left[y_{1},\;y_{2},\;\ldots ,\;y_{N}\right]}^{T}$ (Fig. 2B), where $y_{i}$ is the relative expression at spot location $x_{i}$.

### 2.3 Spatial modeling of gene expression

#### 2.3.1 Homoscedastic model

Due to the effect of tissue niche and intercellular communication, the expression at any spot is regulated by nearby spots. Therefore, the expression at a particular spot has some dependency on the location of the spot, and the covariance of nearby spots is likely to be higher. We model this by decomposing the expression profile *y* into two components—(i) f(X), which captures the spatial dependency of *y*, and (ii) ϵ, the “noise” or the residue term that captures the part of *y* that is not explained by f(X).


(1) y=f(X)+ϵ.

Here, we model f(X) as a sample from a Gaussian process (GP) (Rasmussen and Williams 2005) with a mean function μ(X) and covariance kernel $K(X,X^{\prime })$,


(2) $$f(X)∼GP\left(\mu (X),\;K(X,X^{\prime })\right).$$

For the prior mean function, standard practice is to use a constant mean across spatial coordinates, i.e. $\mu (X)=\mu_{c}$, where $\mu_{c}$ is a constant, because the expression pattern of a gene across different spots is unknown (Rasmussen and Williams 2005, Svensson *et al.* 2018). For the covariance kernel, we chose the radial basis function (RBF) kernel (Rasmussen and Williams 2005), i.e. $K(X,X^{\prime })=\sigma_{k}^{2}\;\text{exp}\;\left(-||X-X^{\prime }|{|}^{2}/2l^{2}\right)$. The RBF kernel is a popular choice for GP regression because it is flexible, can capture nonlinearities in the data, and results in a smooth function f(X) since nearby spots have higher correlation because $||X-X^{\prime }|{|}^{2}$ is lower.

The kernel variance parameter, $\sigma_{k}^{2}$, of the RBF kernel denotes the confidence on the training data and controls the extent of f(X), i.e. how much the samples from *GP* are allowed to deviate from the mean μ(X). The length-scale parameter *l* controls the smoothness of f(X), i.e. how much the expression of two nearby spots of a sample from the *GP* are allowed to deviate from each other. A higher value of *l* results in a smoother and slow changing f(X), while a lower value of *l* results in a more jagged f(X). It should be noted that since we are using different models for each gene, $\mu_{c}$, $\sigma_{k}^{2}$, and *l* are gene-specific parameters.

Since the number of spots is discrete, the GP of Equation (2) boils down to a multivariate Gaussian distribution with mean vector $\mu ={\left[\mu_{c},\;\mu_{c},\;\ldots ,\;\mu_{c}\right]}^{T}$ of length *N* and covariance matrix $\Sigma_{k}$ of size N×N, where the (i,j) th entry is given by the RBF kernel, $\Sigma_{k(i,j)}=\sigma_{k}^{2}\;\text{exp}\;\left(-||x_{i}-x_{j}|{|}^{2}/2l^{2}\right)$, and *N* is the number of spots. Hence, we can write


(3) $$f(X)∼N\left(\mu ,\;\Sigma_{k}\right).$$

The second term, ϵ, in Equation (1) is the “noise” term that captures the part of *y* that is not explained by the RBF kernel, which includes technical noise as well as biological noise. For a regular homoscedastic GP model, we assume the noise to be independent and identically distributed (i.i.d.) as a zero-mean Gaussian with variance $\sigma_{n}^{2}$. Therefore, we can write where $\Sigma_{n}=\sigma_{n}^{2}I_{N}$ is the noise covariance matrix. Combining Equations (1)–(4), we get the likelihood of *y* in terms of the spatial coordinates *X* and the parameters $\mu_{c}$, $\sigma_{k}^{2}$, *l*, and $\sigma_{n}^{2}$,


(4) $$\epsilon ∼N\left(0,\;\Sigma_{n}\right),$$

(5) P(y | X,μc,σk2,l,σn2)=N(μ, Σk+Σn)=N(μ, Σk+σn2IN).

These parameters can be estimated by minimizing the negative log-likelihood [Equation (5)] on a given $N_{T}$ training observations $D={\left\{\left(y_{i},\;x_{i}\right)\right\}}_{i=1}^{N_{T}}$, i.e. $\mu_{c}^{*},\sigma_{k}^{2*},l^{*},\sigma_{n}^{2*}=\arg\;\min\;L_{1}$, where


(6) $$\begin{array}{c} L_{1}=-\text{log}\;P(y\;|\;X,\mu_{c},\sigma_{k}^{2},l,\sigma_{n}^{2})\\ =\frac{1}{2}{\left(y-\mu \right)}^{⊤}{\left(\Sigma_{k}+\sigma_{n}^{2}I_{N}\right)}^{-1}(y-\mu )\\ +\frac{1}{2}\text{log}\;\left|\Sigma_{k}+\sigma_{n}^{2}I_{N}\right|+\frac{N}{2}\text{log}\;2\pi . \end{array}$$

The posterior distribution of *y* can then be calculated by conditioning the prior GP with the calculated parameters and training observations D. This distribution will also be a multivariate Gaussian (Rasmussen and Williams 2005) with mean $\mu_{\text{GP}}$ and covariance $\Sigma_{\text{GP}}$ given by where $\mu^{*}$, $\Sigma_{k}^{*}$, and $\sigma_{n}^{2*}$ are the estimated values of μ, $\Sigma_{k}$, and $\sigma_{n}^{2}$, respectively.


(7) $$\begin{array}{c} \mu_{\text{GP}}=\mu^{*}+\Sigma_{k}^{*}{\left(\Sigma_{k}^{*}+\sigma_{n}^{2*}I_{N}\right)}^{-1}\left(y-\mu^{*}\right) \end{array}$$

(8) $$\Sigma_{\text{GP}}=\Sigma_{k}^{*}-\Sigma_{k}^{*⊤}{\left(\Sigma_{k}^{*}+\sigma_{n}^{2*}I_{N}\right)}^{-1}\Sigma_{k}^{*},$$

#### 2.3.2 Heteroscedastic model

The homoscedastic GP model described above assumes the noise to be independent and identically distributed. However, if the sample being analyzed is heterogeneous, e.g. in terms of cell type, or due to spatially localized pathophysiological processes, the assumption might not hold for some genes. Therefore, we model the noise to have a location-dependent variance $\sigma_{n}^{2}(x_{i})=\sigma_{ni}^{2}$. Hence, assuming the noise variance at location $x_{i}$ is $\sigma_{ni}^{2}$, the noise covariance matrix in Equation (4) becomes a diagonal matrix $\Sigma_{n}=\text{diag}\left(\left[\sigma_{n1}^{2},\;\sigma_{n2}^{2},\;\ldots ,\;\sigma_{nN}^{2}\right]\right)$.

The above noise model where the noise variance is location dependent is called a heteroscedastic noise, and the GP model with heteroscedastic noise is known as a heteroscedastic Gaussian process (HGP). Estimating the noise variance for each location might lead to overfitting and is computationally intractable. Thus a second independent GP is usually used to model the location-dependent noise variance (Le *et al.* 2005, Kersting *et al.* 2007). In our case, we need to model tens of thousands of genes. Hence we adopt an approximate method proposed by Urban *et al.* (2015) which provides a fast way to estimate the posterior mean and variance of the HGP model using two independent GPs. The method is briefly described below.

First, the parameters of a regular homoscedastic GP with noise covariance matrix $\Sigma_{n}=\sigma_{n}^{2}I_{N}$ is estimated by minimizing the negative log-likelihood of Equation (6) on a given training observations D using gradient-based Broyden–Fletcher–Goldfarb–Shanno algorithm (Fletcher 2000). These parameters are then used to calculate the posterior mean $\mu_{\text{GP}}$ and covariance $\Sigma_{\text{GP}}$ using Equations (7) and (8). Next, a new dataset $D_{v}={\left\{z_{i},\;x_{i}\right\}}_{i=1}^{N}$ is formed, where is the difference between the one-sample empirical variance at the *i*th spot and the variance for the *i*th spot. After that, a second homoscedastic GP with constant zero mean and a separate covariance matrix $\Sigma_{kv}$ and a separate noise covariance matrix $\Sigma_{nv}=\sigma_{nv}^{2}I_{N}$ is fitted on the newly formed dataset $D_{v}$ by minimizing the negative log-likelihood where $z={\left[z_{1},\;z_{2},\;\ldots ,\;z_{N}\right]}^{T}$. Finally, denoting the posterior predictive mean of the second GP regression model as $\mu_{\text{GP}v}$, the mean $\mu_{\text{HGP}}$ and variance $\sigma_{\text{HGP}}^{2}$ of the HGP regression model for *y* is estimated by combining the two heteroscedastic GPs according to


$$z_{i}={\left(y_{i}-\mu_{\text{GP},i}\right)}^{2}-\Sigma_{\text{GP}(i,i)}$$

$$\begin{array}{c} L_{2}=\frac{1}{2}z^{⊤}{\left(\Sigma_{kv}+\sigma_{nv}^{2}I_{N}\right)}^{-1}z\\ +\frac{1}{2}\text{log}\;\left|\Sigma_{kv}+\sigma_{nv}^{2}I_{N}\right|+\frac{N}{2}\text{log}\;2\pi , \end{array}$$

$$\begin{array}{c} \mu_{\text{HGP}}=\mu_{\text{GP}}\;\text{and}\\ \sigma_{\text{HGP}}^{2}=\text{diag}\left(\Sigma_{\text{HGP}}\right)=\max\left(0,\;\text{diag}(\Sigma_{\text{GP}})+\mu_{\text{GP}v}\right). \end{array}$$

Note that for this method, both the homoscedastic GP and the (approximate) heteroscedastic GP models have the same mean, and only the variance is refined for the HGP model.

### 2.4 Statistical test to detect noisy genes

To determine which model yields a better fit, we compare the NLPD of the regular homoscedastic model ${\text{NLPD}}_{\text{Homo}}$ with that of the heteroscedastic model ${\text{NLPD}}_{\text{Hetero}}$. The NLPDs on a validation dataset $D_{T}={\left\{\left(y_{j}^{\prime },\;x_{j}^{\prime }\right)\right\}}_{j=1}^{N_{T}^{\prime }}$ for the two models are calculated using the equation where $\mu_{T^{\prime },i}$ and $\sigma_{T^{\prime },i}^{2}$ are the predicted mean and variance of the models, respectively, at the *i*th spot on the validation dataset $D_{T^{\prime }}$, and $N^{\prime }$ is the number of spots in each test sample $\left\{y_{j}^{\prime },\;x_{j}^{\prime }\right\}$.


(9) $$\begin{array}{c} \text{NLPD}=-\frac{1}{N^{\prime }}\sum_{i=1}^{N^{\prime }}\;\text{log}\;N\left(y_{i}^{\prime }\;|\;\mu_{T^{\prime },i},\;\sigma_{T^{\prime },i}^{2}\right)\\ =\frac{1}{N^{\prime }}\sum_{i=1}^{N^{\prime }}\left(\text{log}\;2\pi \sigma_{T^{\prime },i}^{2}+\frac{{\left(y_{i}^{\prime }-\mu_{T^{\prime },i}\right)}^{2}}{2\sigma_{T^{\prime },i}^{2}}\right), \end{array}$$

A statistical test is required to assess whether the difference between the NLPDs of the two models is significant. However, as the background distribution of this difference is unknown, we cannot use a *z*-test or *t*-test because they assume a normal distribution (Sprinthall 2013). Therefore, we resort to a nonparametric test. Specifically, Wilcoxon signed-rank test (Woolson 2008) was used on a set of paired NLPD values obtained by repeating the model fitting process 10 times with a different random splitting of the spots into 90% training and 10% validation set. We also used the Benjamini–Hochberg method (Benjamini and Hochberg 1995) to correct the false discovery rate (FDR). This bootstrapping procedure helps in reducing the overfitting of the model to the training set and also provides a more robust model comparison. If the NLPD of the heteroscedastic GP model is statistically significantly lower than that of the regular GP model, then the gene is considered to have location-dependent noise variance and is denoted as the *noisy* gene in this article.

### 2.5 Clustering

The goal of the clustering algorithm is to put two *noisy* genes in the same cluster if they show similar location-dependent noise variance patterns. However, this cannot be done by simply taking the $L_{2}$ distance of the noise variances, because they differ in scale. For this, we defined a heuristic distance function between the predicted noise variance of two genes. To calculate this distance, first, the predicted noise variances for the genes are binarized, i.e. converted to 0 or 1, by comparing the values to a threshold obtained from Otsu’s method (Otsu 1979) (Supplementary Fig. S1A). Next, the intersection-over-union (IoU) [also known as the Jaccard similarity index (Jaccard 1912)], $J_{I}$, is calculated between the binarized gene variance. Finally, the distance between the variance of the two genes is defined as $J_{D}=1-J_{I}$. A low value of $J_{D}$ between two genes indicates that the two genes show high noise variance in similar spatial regions (Supplementary Fig. S1B). Bottom-up (agglomerative) hierarchical clustering (Rokach and Maimon 2005) is then performed using this $J_{D}$ as the distance metric (Supplementary Fig. S1C). The cluster representative is calculated as the average gene expression variance (averaged over cluster members) (Supplementary Fig. S1D).

### 2.6 Cell abundance and cell type heterogeneity estimation for the Visium data

For Visium data, each spot contains 1–10 cells, but the exact number of cells in each spot is not known. However, the total number of cells in a spot tightly corresponds to the total transcript count of all the genes expressed in that spot (Gulati *et al.* 2020, Vahid *et al.* 2023). Thus, we use this total transcript count in each spot as a proxy measurement of cell abundance distribution for the squamous cell carcinoma Visium data.

To find out where different cell types are located in a tissue, we follow an approach similar to Tirosh *et al.* (2016) and Dries *et al.* (2021). This approach involves first finding the marker genes for different cell types, and then calculating the enrichment of these marker genes. The scRNA-seq data from the same tissue slice as the Visium data used in this study contains cell-type annotations of each cell. This information was used to find the marker genes for each cell type by applying the FindAllMarkers function of Seurat (Satija *et al.* 2015) on the annotations to obtain the differentially expressed genes for every cell type. The top 20 differentially expressed genes (in terms of the log-fold change) for each cell type were taken as its marker.

To measure the enrichment of a given cell type at a Visium spot, we first calculate the average expression of the marker gene set for that spot and denote this as the program score. Next, we calculate the average expressions of 1000 control gene sets and denote them as control scores. A control gene set is defined by first binning all the genes into 50 bins of aggregated expression levels across all spots and then, for each gene in the marker gene set, randomly selecting a gene from the same expression bin as that gene. Finally, we count the number of times the control scores were greater than the program score and obtain a *P*-value by dividing this count by 1000. We denote the spot to be enriched by that cell type if the *P*-value is <.05. The total number of cell types at each spot is used as a measure of cell-type spatial heterogeneity.

### 2.7 Explanatory variable analysis

As discussed earlier, the heteroscedasticity of gene expression can be caused by factors like cell-type variation, cell abundance variation, spatially localized pathophysiological processes, etc. We refer to such possible factors underlying heteroscedasticity as *explanatory variables*. To identify the extent of *noisy* genes caused by a given explanatory variable, we performed bivariate analyses (Babbie 2021) between the predicted noise variances of the *noisy* genes and the explanatory variable. Specifically, we calculated the Spearman correlation coefficient (ρ) between the predicted noise variances of the detected *noisy* genes and the explanatory variable. The noisy genes caused by the explanatory variable are then selected as those that have $\left|\rho \right|>\rho_{\text{threshold}}$ at an FDR of 0.05.

## 3 Results

### 3.1 NoVaTeST: a pipeline for detecting genes with spatially variable noise

NoVaTeST can detect genes with location-dependent noise variance by comparing the NLPD of a model with constant noise to a model with spatially variable noise. After an initial quality check (QC) filtering, the method first uses a regular GP and a heteroscedastic GP to model the normalized and variance-stabilized gene expression profile of a gene as a function of spatial location. For each gene, the two models are evaluated 10 times. We take a random split of the data (90% train, 10% validation) every time, train the models, and record their NLPD values on the validation data. We then compare these 10 pairwise NLPD values using Wilcoxon signed-rank test, perform FDR correction, and call a gene to be *noisy* if the FDR-corrected *P*-value is <.05. These *noisy* genes are then clustered based on the similarity of their location-dependent noise variance patterns. This process can subsequently be followed by a bivariate analysis to check if a given explanatory variable, e.g. cell-type abundance, can explain the heteroscedasticity of one or more *noisy* genes.

### 3.2 NoVaTeST detects genes enriched in cancer-related pathways in squamous cell carcinoma data

**Figure 3:**
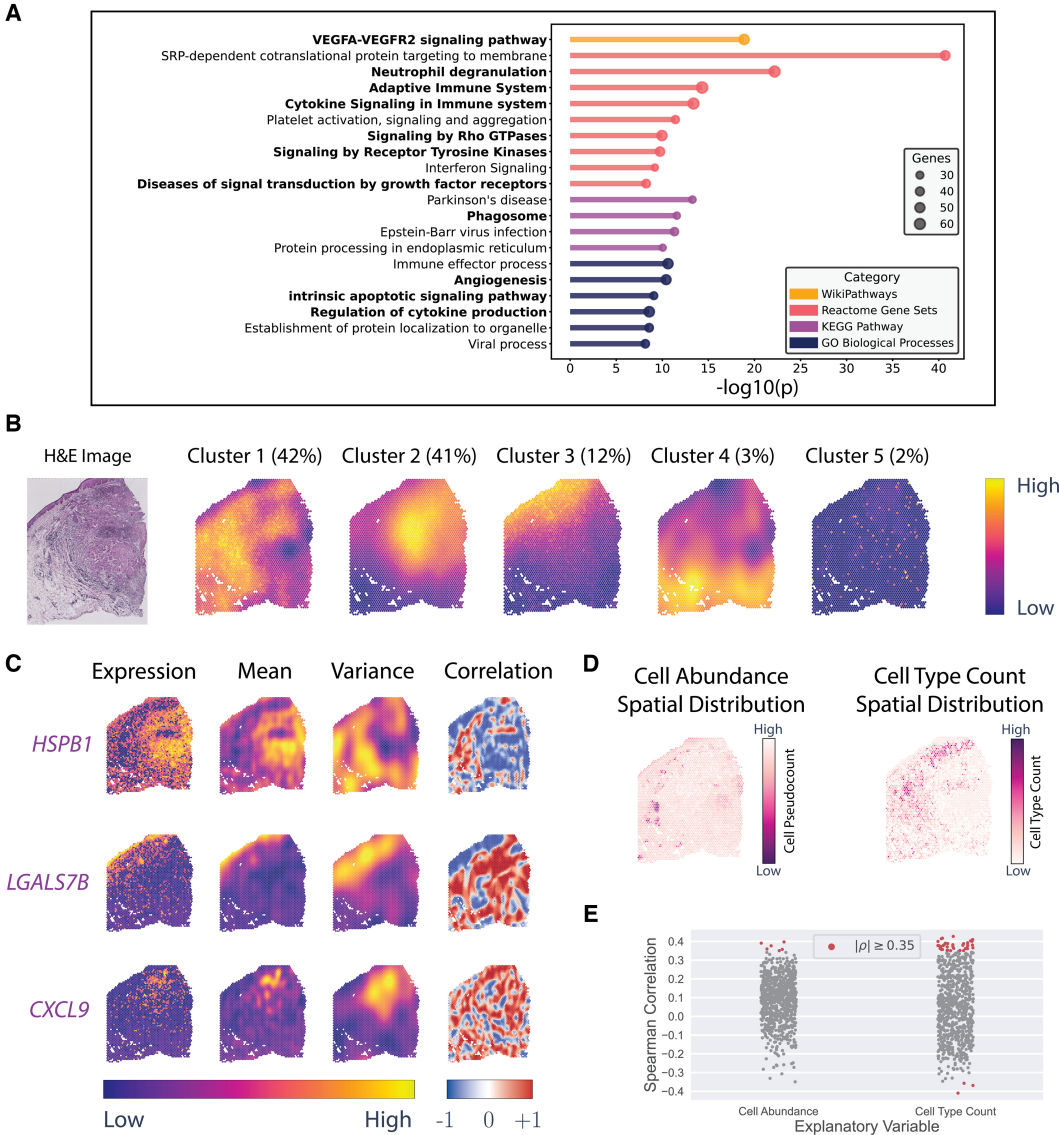
Results obtained from squamous cell carcinoma data using NoVaTeST. (A) Top cluster representative enriched terms for the detected *noisy* genes using Metascape. Terms associated with cancer and immuno-response are shown in bold font. (B) The average gene expression noise variance, averaged over the cluster members, for the five identified clusters along with the tissue H&E-stained image. The numbers inside the parentheses denote the percentage of *noisy* genes in each cluster. (C) Gene expression (log scale) and corresponding modeled mean and variance for three representative genes from the first three clusters. Also plotted is the spatial Spearman correlation between the mean and variance of the model, where the correlation for a spot is computed by considering 12 nearby spots. (D) Spatial distribution of two possible explanatory variables for the detected *noisy* genes, namely cell abundance and cell type count variation across spatial coordinates. (E) Spearman correlation of the bivariate analysis of the *noisy* genes with the two possible explanatory variables.

Applying our model on the first dataset with squamous cell carcinoma cells and patient-matched adjacent normal cells (Ji *et al.* 2020), we identified 771 *noisy* genes at an FDR level of 5% (Supplementary Table S1). We performed enrichment analysis of the *noisy* genes to detect all statistically significant enrichment terms and hierarchically clustered them into a tree based on the similarity of their gene memberships using Metascape (Zhou *et al.* 2019). The resulting top cluster representative enrichment terms are shown in Fig. 3A. The terms associated with cancer and immuno-response are marked bold in Fig. 3A. Interestingly, the *noisy* genes for this dataset are mostly associated with cancer-related pathways—not only cancer development (Mollinedo 2019), tumor progression (Lee and Rhee 2017, Yamazaki *et al.* 2020), angiogenesis (Abhinand *et al.* 2016), but also cancer immuno-response (Fulda and Debatin 2006). This result makes sense since tumor microenvironment are highly heterogeneous due to their uncontrolled growth and thus the genes related to cancer and immuno-response are likely to have different noise variance in the tumor region compared to the tumor-adjacent healthy region.

The *noisy* genes were clustered based on a heuristic approach (details provided in Section 2) to find groups of genes that show visually similar noise-variance patterns. The cluster representative, which is the gene expression noise variance averaged over cluster members, for the five identified clusters in the *noisy* genes are shown in Fig. 3B. The distribution of the 771 *noisy* genes, shown beside the cluster labels in Fig. 3B, reveals that most of the genes belong to clusters 1, 2, and 3 (Supplementary Table S1). Comparing the cluster representatives and the tissue H&E-stained image, we see that for cluster 1, the variance is high mainly in the stroma and tumor-adjacent healthy region, whereas, for cluster 2, the variance is high mainly in the tumor region. For cluster 3, the variance is high along the thin line on the upper-left of the H&E image, which corresponds to the benign squamous epithelium and stroma region (see Fig. 4D of Erickson et al. 2022).

**Figure 4:**
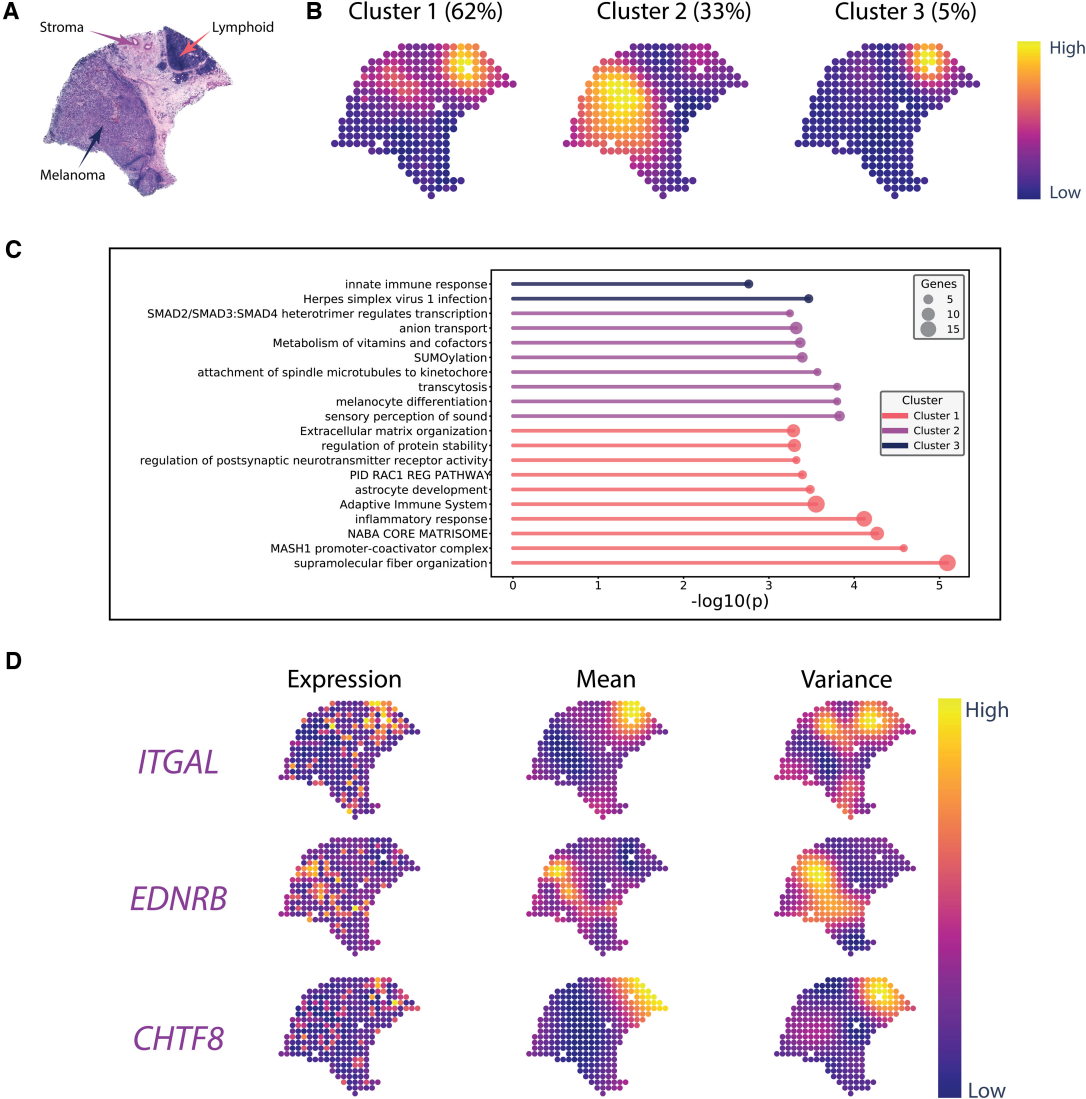
Results obtained from cutaneous malignant melanoma data using NoVaTeST. (A) Tissue H&E-stained image of the sample with three histopathological regions—melanoma, lymphoid, and stroma. (B) The average gene expression noise variance, averaged over the cluster members, for the three identified clusters. The numbers inside the parentheses denote the percentage of *noisy* genes in each cluster. (C) Top cluster representative enriched terms for the detected *noisy* genes in each detected cluster using Metascape. (D) Gene expression (log scale) and corresponding modeled mean and variance for three representative genes from the three clusters.

The log-expression and the mean and variance of the heteroscedastic model for three representative genes, *HSPB1*, *LGALS7B*, and *CXCL9*, selected from cluster 1, 2, and 3, respectively, are shown in Fig. 3D. *HSPB1*, which shows high mean in the tumor region and high variance primarily in the adjacent healthy and the stroma region of the H&E image, is strongly associated with tumor metastasis and metastatic colonization (Blackburn *et al.* 1997, Lemieux *et al.* 1998, Bausero *et al.* 2004, Gibert *et al.* 2012, Nagaraja *et al.* 2012). *LGALS7B* is known to play role in several types of carcinomas (Fujimoto *et al.* 2013), and the expression shows a high mean in the squamous epithelium region but a high variance in the stroma region. *CXCL9* from cluster 2 shows high variance in the tumor-affected region and is involved in T-cell trafficking (Ochiai *et al.* 2015). These results indicate that genes with significant spatially variable noise detected by our method indeed carry biologically significant results, and thus the importance of a generalized model.

For the selected genes mentioned earlier, we calculated the spatial correlation between the estimated mean and variance by calculating the Spearman correlation over the 12 nearest neighbors of each spot. The results are shown in Fig. 3C. For *HSPB1*, we see that the correlation is overall low for almost all the spots. For *LGALS7B*, on the other hand, the correlation is low for the spots where the variance is high (and vice versa). These results are indications that these genes are not an artifact of the mean–variance relationship, and thus provide complementary information to existing methods (see Section 4).

Next, we calculate the spatial heterogeneity of cell abundance and cell type of the sample (Fig. 3D) to check if they can explain the heteroscedasticity of the detected *noisy* genes. The results of the bivariate analyses between the two explanatory variables and the variance of the *noisy* genes for a threshold of $\rho_{\text{threshold}}=0.35$ are shown in Fig. 3E. At this threshold, we find that the cell abundance variation is moderately correlated with the variance of 6 *noisy* genes, while cell-type heterogeneity is moderately correlated with the variance of 42 *noisy* genes. This means that cell-type heterogeneity and cell abundance variation could be the probable causes of those 6 and 42 *noisy* genes, respectively. Interestingly, 39 of the 42 *noisy* genes moderately correlated with cell-type heterogeneity belong to cluster 1.

### 3.3 NoVaTeST identifies distinct noise variance patterns in cutaneous malignant melanoma data

The second dataset contains the expression of 13 088 genes expressed across 279 spots from a melanoma lymph node biopsy sample collected using spatially resolved transcriptomics technology (Thrane *et al.* 2018). The three main regions in the tissue H&E-stained histopathology image, namely, melanoma, stroma, and lymphoid, are pointed out in Fig. 4A. The high-resolution histology image was provided by the authors of the dataset. A detailed annotation by an expert pathologist of an adjacent tissue section is available in the Supplementary Figure S1 of the original publication (Thrane *et al.* 2018).

Applying our model, we find 473 *noisy* genes at an FDR level of 5% (Supplementary Table S2). We first clustered the *noisy* genes into three clusters based on the proposed heuristic approach to elucidate the biological processes impacted by these genes (Supplementary Table S2). The gene expression noise variances averaged over cluster members for the three identified clusters are shown in Fig. 4B. The high variance regions of the cluster representatives of clusters 1 and 3 overlap with the manually annotated lymphoid regions of the H&E image. On the other hand, genes in cluster 2 show high variance in the melanoma region. These results point to the importance of the heteroscedastic model, as variance in phenotypically different regions shows different patterns of noise variance.

Next, enrichment analysis was performed for each cluster to identify the top enriched terms. Then, the top enriched terms were hierarchically clustered based on gene memberships’ similarity. The top enriched cluster representative terms for the melanoma dataset across the three identified clusters are shown in Fig. 4C. Notably, genes in cluster 1, which show high variance in the lymphoid region, are enriched in “supramolecular fiber organization,” as well as immuno-response-related terms “inflammatory response” and “adaptive immune response.” Genes in cluster 3, which also show high variance in the lymphoid region, result in only two clustered enriched terms (as there are only 24 genes), one of which is “innate immune response.” Lastly, genes in cluster 2 show high noise variance in the melanoma region, and are enriched in the GO term “melanocyte differentiation.” These results indicate that genes with similar noise-variance patterns might perform similar operations.

The log-expression and the mean and variance of the heteroscedastic model for three representative genes, *ITGAL*, *EDNRA*, and *CHTF8*, selected from clusters 1, 2, and 3, respectively, are shown in Fig. 4D. Abnormal expression of *ITGAL* is linked with immune regulation (Zhang *et al.* 2022). High expression of *ENDRB* is linked with melanoma metastases (Saldana-Caboverde and Kos 2010), which in this sample is also the region where the variance is high. The results indicate that the heteroscedastic model can be used to identify genes with abnormal expression in specific regions of the tissue.

## 4 Discussion

An important first step for ST data analysis is modeling the gene expression as a function of location. There are two main types of uncertainty to consider while modeling, namely *epistemic* uncertainty and *aleatoric* uncertainty. The epistemic uncertainty is the variability of the model output due to the randomness of the model itself. In the case of modeling ST data, this refers to the uncertainty (or confidence) of gene expression prediction given spatial location. On the other hand, the aleatoric uncertainty is the variability of the model output due to the randomness or noise present in the data. In this case, aleatoric variability is the part of gene expression that have no spatial variation, also referred to as noise. The aleatoric uncertainty can be further classified into *homoscedastic* or input-independent constant noise, and *heteroscedastic* or input-dependent noise.

While existing methods use a Bayesian framework to capture the epistemic uncertainty, they use a constrained assumption of homoscedastic noise to model the aleatoric uncertainty. The proposed NoVaTeST pipeline can detect the presence of heteroscedastic uncertainty, i.e. genes that show significant spatial variation in noise variance.

**Figure 5:**
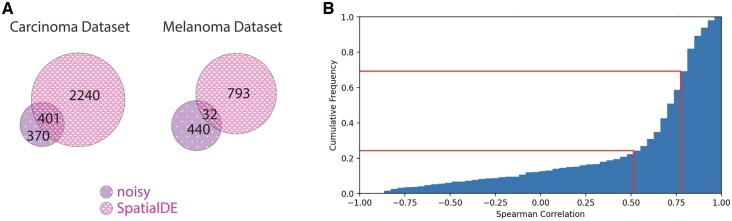
Analysis to check the existence and extent of mean–variance artifacts in the datasets. (A) Venn diagram showing the overlap between genes detected by SpatialDE and NoVaTeST for the carcinoma and the melanoma datasets. (B) Cumulative frequency of Spearman correlation between the estimated mean and variance for the common genes (genes detected by both SpatialDE and NoVaTeST).

To check whether the *noisy* genes are an artifact of the mean–variance relationship, we compared the *noisy* gene list to that detected by SpatialDE (Svensson *et al.* 2018), a tool to detect genes with significant spatial mean expression patterns. If the detected *noisy* genes were indeed an artifact, then the *noisy* genes would also have been detected by SpatialDE. However, only about 50% of the *noisy* genes from the carcinoma data were common with the 2641 genes detected by SpatialDE (Fig. 5A). The rest 370 *noisy* genes were not detected by SpatialDE, meaning the expressions of these genes do not show any significant spatial pattern, but their noise-variances display a location-dependent pattern. For the melanoma dataset, only 32 out of the 470 *noisy* genes overlap with the 825 genes detected by SpatialDE (Fig. 5A). These results demonstrate that the detected noisy genes are not an artifact of the mean–variance relation, rather they provide complementary information to existing methods like SpatialDE.

Further analysis, however, reveals that the detected *noisy* genes are not completely unaffected by the mean–variance artifact, especially the *overlapping* genes detected by both NoVaTeST and SpatialDE. This can be seen from the cumulative histogram of Spearman correlation between the mean and the variance of the 401 *overlapping* genes of the carcinoma dataset (Fig. 5B). Around 50% or 200 genes show moderate correlation (between 0.5 and 0.75, as marked by the two red lines in Fig. 5B), meaning the mean and variance are somewhat correlated, i.e. spots with high expression have high variance. On top of that, around 25% or 100 genes show a strong correlation between mean and variance at each spot. These genes could be an artifact of the mean–variance relation, thus suggesting a more robust variance-stabilizing transformation should be adopted.

For a sample with *N* spots, each GP model has a time complexity of $O(N^{3})$ and space complexity of $O(N^{2})$. Thus, with *G* genes expressed across *N* spots, the total time complexity for the GP modeling is $O(GN^{3})$ and space complexity is $O(GN^{2})$. The approximate heteroscedastic model in NoVaTeST uses two regular GPs to capture the location-dependent noise. Thus the time and space complexity remains $O(GN^{3})$ and $O(GN^{2})$, respectively.

## 5 Conclusion

In this article, we have proposed the NoVaTeST pipeline that offers a more general spatial gene expression modeling in ST data using the HGP. The pipeline uses Wilcoxon signed-rank test and FDR correction to identify genes with location-dependent noise variance. Analysis of squamous cell carcinoma ST data shows that the detected *noisy* genes (genes that show significant heteroscedastic noise) are mostly associated with cancer and immuno-response-related pathways. On the other hand, the noisy genes in cutaneous malignant melanoma data form three clusters in terms of noise-variance pattern, and these patterns overlap with manual annotation of different phenotypical conditions in the H&E image. These results are consistent with our initial hypothesis regarding the noisy genes and provide evidence of the biological significance of the *noisy* genes. Moreover, we have shown that the pipeline provides complementary information to existing techniques such as SpatialDE. Our work opens up several directions for future research. First, as ST data contain information on cell–cell communications, we want to investigate whether the noise variance patterns and the detected noisy genes reveal any novel information regarding cell–cell communications. Second, we want to extend the model to other spatial data modalities such as spatial proteomics, epigenetics, and metabolomics. Finally, we want to improve the time complexity of the current GP models used for ST data to assure efficient execution at the level of whole human tissues.

## Supplementary Material

btad372_Supplementary_Data
