# Supplementary material for: NoVaTeST: identifying genes with location-dependent noise variance in spatial transcriptomics data
Source: Bioinformatics. 2023 Jun 7;39(6):btad372. doi: 10.1093/bioinformatics/btad372 (PMC10283152; doi:10.1093/bioinformatics/btad372)
Supplement: btad372_Supplementary_Data [file btad372_supplementary_data.zip › suppl_data/Supplementary_Figure_S1.pdf]

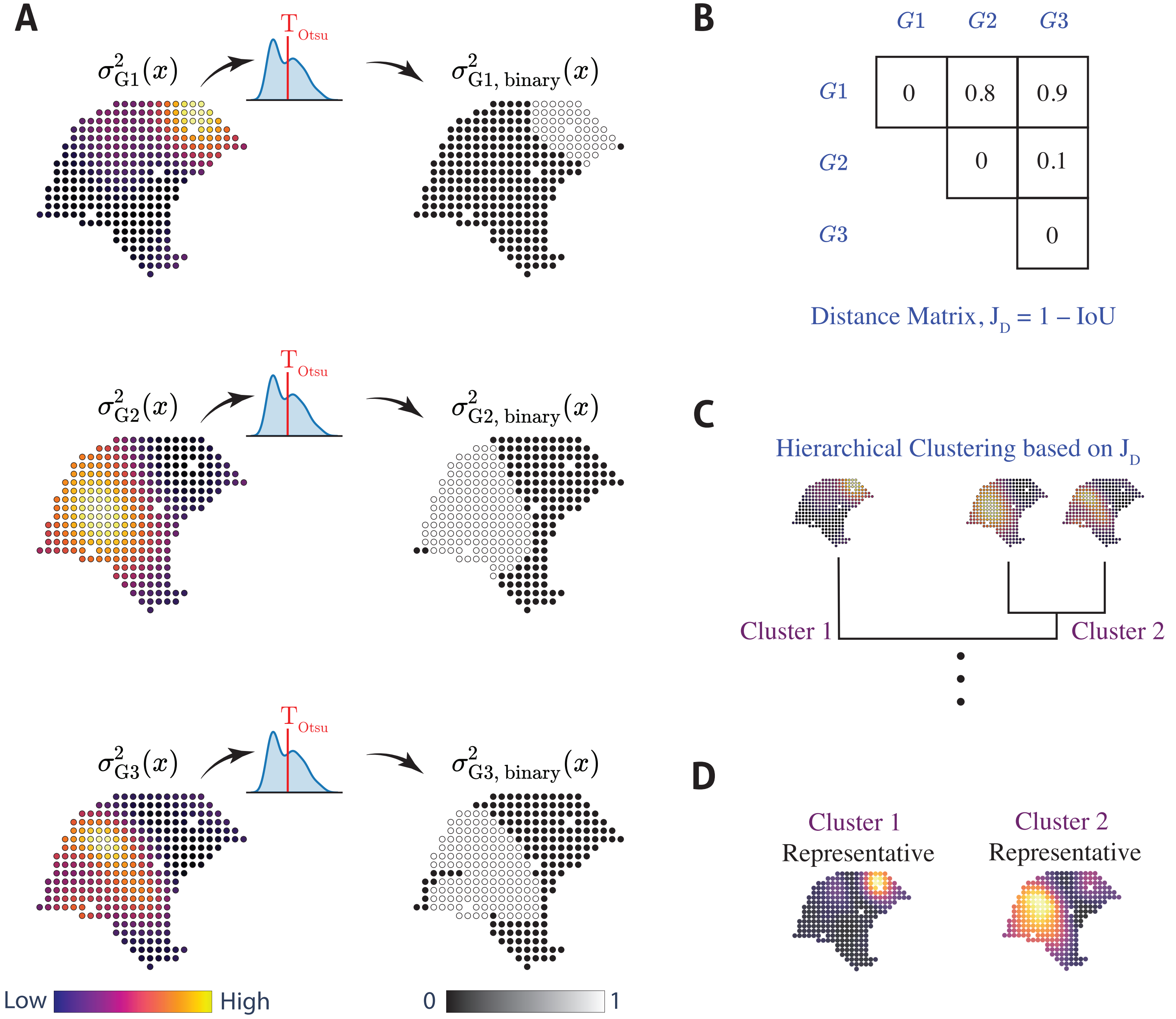

Supplementary Figure S1: Brief overview of the clustering method. (**A**) The predicted noise variance of the *noisy genes* are first binarized by comparing them to the corresponding Otsu's threshold. (**B**) Intersection over Union (IoU) index is used to calculate the distance between binarized variances between two genes, which is defined as $J_D = 1 - \text{IoU}$. Pairwise distances are used to form the distance matrix. (**C**) Agglomerative hierarchical clustering is used to cluster the genes based on the distance matrix. (**D**) Cluster representatives are calculated as the average variance across all cluster members.
